# Azoxystrobin induces apoptosis *via* PI3K/AKT and MAPK signal pathways in oral leukoplakia progression

**DOI:** 10.3389/fphar.2022.912084

**Published:** 2022-08-04

**Authors:** Lingyu Li, Jing Li, Hui Chen, Yajun Shen, Yunping Lu, Min Zhang, Xiaofei Tang

**Affiliations:** ^1^ Division of Oral Pathology, Beijing Stomatological Hospital and School of Stomatology, Beijing Institute of Dental Research, Capital Medical University, Beijing, China; ^2^ Department of stomatology, Beijing Friendship Hospital, Capital Medical University, Beijing, China

**Keywords:** azoxystrobin, oral leukoplakia, apoptosis, network pharmacology, bioinformatics

## Abstract

**Background:** Oral leukoplakia (OLK) is one of the oral potentially malignant disorders (OPMDs) with an increased risk of developing oral squamous cell carcinoma (OSCC). There is no ideal therapeutic drug yet. Our previous study showed azoxystrobin (AZOX) inhibited the viability of OLK cells and the incidence of mouse tongue cancer. However, its specific mechanism has not been clarified. Here, we used network pharmacology with experimental validation to investigate the roles and mechanisms of AZOX in OLK.

**Methods:** The targets of AZOX and OLK were obtained from online databases. The overlapping genes were identified by the Jvenn database. STRING and Cytoscape software were used to construct the PPI network. GO and KEGG enrichment analyses were used to analyze the biological function. Molecular docking and CETSA were used to verify the direct binding between AZOX and its key targets. 4NQO induced mouse tongue carcinogenesis model was constructed to clarify the treatment response of AZOX *in vivo*. TUNEL staining was performed to detect the effect of AZOX on apoptosis in mouse OLK tissues. CCK8 assay, flow cytometry, and western blot were used to detect the effect of AZOX on cell proliferation and apoptosis in DOK cells. The expression of PI3K/AKT and MAPK markers were analyzed by immunohistochemistry *in vivo* or by western blot *in vitro*.

**Results:** Venn diagram showed 457 overlapping targets, which were involved in the PI3K/AKT, MAPK, and apoptosis pathways, and the top 5 hub modules were TP53, STAT3, AKT1, MAPK1, and PIK3R1. AZOX was bound with the highest force to AKT and PI3K by AutoDock Vina. PyMOL software visualized that AZOX could fit in the binding pocket of the AKT and PI3K. The carcinogenesis rate of the mouse OLK in the high-dose AZOX group was significantly reduced. AZOX induced apoptosis in the OLK tissues and DOK cells, and the expression of PI3K, AKT, p-ERK was decreased, and the expression of p-p38 and p-JNK was increased. CETSA indicated that AZOX might have a direct binding with AKT and PI3K.

**Conclusion:** AZOX may induce apoptosis via PI3K/AKT and MAPK pathways in OLK. This study reveals the potential therapeutic targets of AZOX in OLK.

## Introduction

Oral leukoplakia (OLK) is one of the oral potentially malignant disorders (OPMDs) and has the potential to develop into oral squamous cell carcinoma (OSCC) (Warnakulasuriya et al., 2021). A systematic review of observational studies on OLK reported a malignant transformation from OLK to OSCC rate ranging from 0.13 to 34.0% (Ramos-Garcia et al., 2021). The standard of care for OLK is to surgically remove the lesion, if possible (Greenslade, 2017). After surgically removing the lesion, the recurrence rates still range from 10 to 45%, depending on the type of lesion and surgical methods used (Brennan et al., 2007; Sundberg et al., 2019). Furthermore, complete removal of the lesion does not eliminate the risk of carcinogenesis (Holmstrup et al., 2006; Brouns et al., 2014; de Pauli Paglioni et al., 2020). Chemoprevention trials on OLK play an essential role, but the existing drugs are highly unsatisfactory because of the toxicity, mode of administration, and efficacy (Greenslade, 2017).

Azoxystrobin (AZOX), also called Amisida, isolated initially from mushrooms, is a natural insecticide. AZOX is widely used in agriculture and aquaculture to prevent and treat fungal diseases. No carcinogenic, mutagenic, or teratogenic potential effect has been reported (Rodrigues et al., 2013; Iqbal et al., 2018). An AZOX human health risk assessment conducted by the US Environmental Protection Agency showed that no developmental effects were seen in rabbit or rat developmental toxicity studies. And no reproductive or offspring effects were seen in the 2-generation rat reproduction study. However, it was shown that AZOX reduced neuronal viability, neurite outgrowth, and cortical migration process in developing brains (Kang et al., 2021). Studies have shown that AZOX can target mitochondria and inhibit the electron transport of the oxidative phosphorylation process, leading to reduced cellular energy (Huttemann et al., 2007; Shi et al., 2017). The relevant study has shown that AZOX inhibits the viability of human esophageal cancer cells KYSE-150 and induces mitochondrial pathway of apoptosis (Shi et al., 2017). And AZOX inhibits neuronal migration and induces apoptosis in cortical neurons (Kang et al., 2021). In our previous study, we found that AZOX inhibited oral carcinogenesis by specifically targeting mitochondrial complex III, leading to ROS accumulation, MMP reduction and apoptosis. AZOX may be a candidate agent for the prevention and treatment of OSCC. However, the effects and mechanisms of AZOX in OLK are unknown.

Network pharmacology provides a systematic understanding of network theory and systems biology, which is used to study the interaction among drugs, proteins or genes, and diseases. Moreover, it can describe intricacies among biological systems, drugs, and diseases from a network perspective (Zhou et al., 2016).

Here, we established the target library to screen out the targets of AZOX against OLK and analyze the interaction between AZOX and the targets. Further, *in vivo* and *in vitro* studies were used to verify the effects of AZOX on the key targets and investigate its molecular mechanisms in OLK, which provides insights into the potential targets of AZOX in the treatment of OLK and establishes the foundation for its future clinical applications.

## Materials and methods

### Establishment of the target library

To retrieve the targets of AZOX, we obtained the 2D structure of AZOX from PubChem (https://pubchem.ncbi.nlm.nih.gov/). There are many databases available for the target screening of small molecule compounds. The pharmmapper online database (http://www.lilab-ecust.cn/pharmmapper/) is an online website for identifying potential target candidates for the given small molecules using pharmacophore mapping approach. Swiss target prediction database (http://www.swisstargetprediction.ch/) predicts the targets of small molecules based on similarities to the 2D and 3D structures through reverse screening. Comparative toxicogenomics database (CTD; http://ctdbase.org/) is an innovative digital knowledgebase that integrates toxicological information on chemicals, genes, phenotypes, diseases based on literature, manually curated interactions, providing great convenience for studying disease-associated environmental exposure factors and potential mechanisms of drug. The databases described above were used to search for AZOX targets for the present study. Online mendelian inheritance in man (OMIM; https://omim.org/) is a comprehensive and authoritative compendium of human genetic and hereditary phenotypes, focusing on the relationship between phenotypes and genotypes. GeneCards (https://www.genecards.org/) is a comprehensive and user-friendly searchable database of integrated information on genomic, transcriptomic, proteomic, genetic, clinical, and functional information. OMIM, GeneCards, and CTD were used to explore the therapeutic targets related to OLK, and “oral leukoplakia” was used for searching its targets. Finally, the two target sets were collected by the Jvenn database (http://jvenn.toulouse.inra.fr/app/example.html), and the intersection was taken as the target library of this study.

### Construction of protein-protein interaction network

STRING (https://string-db.org/) was used to predict interactions between proteins. The overlapping proteins were uploaded, and the study species were selected as Homo sapiens. We downloaded the protein interaction in TSV format and imported it into the Cytoscape software. The protein-protein interaction (PPI) network was constructed, and the CytoHubba tool was used to analyze the top 10 core regulatory targets of the PPI network.

### GO and KEGG pathway enrichment

GO and KEGG pathway enrichment were performed by R packages, including “clusterProfiler”, “org.Hs.eg.db”, “DOSE” and “pathview”. The top 15 GO terms and KEGG pathways were visualized as bubble plots and bar charts.

### Molecular docking

The 3D structure of the AZOX was obtained from the PubChem database and dealt with ChemDraw software. The PDB format of candidate targets was downloaded from the PDB (https://www.omicshare.com) database. Then, the ligands, non-protein molecules, and water in proteins were removed by Discovery Studio 4.5 software. The AutoDockTools 1.5.6 software was used to prepare AZOX and the target proteins. Molecular docking was performed using AutoDock Vina. After calculations, the top ten highest-scored poses were returned as a docking result. The obtained results were ranked according to their mean score values of the top ten docking scores and presented in kcal/mol. The 3D-interaction diagrams of AZOX and its target proteins were visualized using the PyMOL software.

### Experimental validation

#### Chemicals and reagents

AZOX was purchased from Sigma-Aldrich (MO, United States) and dissolved in dimethyl sulfoxide (DMSO) to form the stock solution of 10 mg/ml. For cell experiments, the solution was diluted with a cell culture medium to different concentrations of AZOX solutions. The final concentration of DMSO in the medium was <0.2% and had a minor effect on cell growth. While for animal experiments, the solution was diluted with corn oil to prepare high-dose (25 mg/kg) and low-dose (5 mg/kg) AZOX solutions. 4-nitro-quinoline-1-oxide (4NQO) was obtained from Sigma-Aldrich (MO, United States) and dissolved in 1,2-propanediol to form the stock solution of 5 mg/ml. This stock solution was then diluted to a concentration of 50 μg/ml with purified water.

#### Animal experiments

The animal experiments were conducted in accordance with the Animal Ethical and Welfare Committee of Beijing Stomatological Hospital (Approval No. KQYY-201,804-010). Thirty-four C57BL/6 mice (8 weeks old, 16-20 g, either sex) were purchased from Beijing Vital River Laboratory Animal Technology Co. (Beijing, China). Mice were randomly grouped as follows. The group without any treatment was used as a blank control group (*n* = 4). The high-dose AZOX group (*n* = 10), low-dose AZOX group (*n* = 10), and vehicle control group (*n* = 10) were administered 50 μg/ml 4NQO water for 10 weeks. Then the high-dose (25 mg/kg) AZOX group and the low-dose (5 mg/kg) AZOX group were intragastric administration of AZOX every other day. The vehicle control group was intragastric administration of corn oil. The mice were euthanized at the end of the 24th week, and their tongues were fixed in 10% neutral formaldehyde for histological examination.

#### Hematoxylin and eosin staining

Five-micron thick paraffin sections were deparaffinized, rehydrated, and strained with hematoxylin and eosin (H&E) routinely. The criteria used in determining OLK were according to the “2017 WHO Classification of Head and Neck Tumours”. Briefly, “Leukoplakia” is a clinical term used to describe white plaques of questionable risk, once other specific conditions and other OPMDs have been ruled out. The diagnosis was verified using pathologic examination. Traditionally, OLK is often accompanied by oral epithelial dysplasia. Judging the number of thirds of the epithelium affected is one factor in defining a grade. Mild dysplasia can be defined by cytological atypia limited to the basal third, moderate dysplasia by extension into the middle third, and severe dysplasia by extension into the upper third. However, architectural and cytological atypia and the architecture of the connective tissue interface should increase the grade. OSCC exhibits distinctive non-keratinizing morphology or differentiated squamous features and arises from crypt epithelium and grows beneath the surface epithelial lining as nests and lobules, often with central necrosis. The staining results were judged by two independent professional pathologists. And the carcinogenesis rate = (number of cancerous lesions/total number of the group) × 100%.

#### TUNEL assay

TUNEL assay for tongue tissues was conducted using a one-step TUNEL cell apoptosis assay kit (red fluorescence) (Beyotime, China), according to the kit instructions. Nuclei were marked by DAPI. A fluorescence microscope (Olympus BX61, Japan) was used for observation. Apoptotic cells were characterized by co-localization of the TUNEL label (red) and DAPI (blue), and the relative apoptosis rate was calculated using ImageJ software.

#### Immunohistochemistry

Immunohistochemistry (IHC) was performed as described previously (Chen et al., 2020). Briefly, sections were stained with the PI3CA (1:300, A0265, Abclonal), AKT antibody (1:300, 10176-2-AP, ProteinTech), p-ERK (1:200, #4370, Cell Signaling Technology), p-JNK (1:20, #4668, Cell Signaling Technology), p-p38 (1:20, #4631, Cell Signaling Technology) antibody. Stained slides were observed with a light microscope at a 200 × objective. Three visual fields in each section were used to determine the mean and standard error. The IHC images were calculated using Image-Pro Plus software 6.0.

#### Cell culture

The dysplastic oral keratinocyte (DOK) cells (ECACC 94122104, purchased from the European Collection of Authenticated Cell Cultures data bank) were gifted by Dr. Qianming Chen (West China Medical center, China). The DOK cells were cultured in high-glucose Dulbecco’s modified Eagle’s medium (Gibco, United States) containing 10% fetal bovine serum (Gibco, United States), 1% penicillin/streptomycin (Biosharp, China), and 5 μg/ml hydrocortisone (Beyotime, China). Cells were incubated at 37°C under 5% CO_2_ in a humidified atmosphere.

#### CCK8

The cell viability was performed with a CCK8 kit (Dojindo, Japan) following the producer’s suggestions. Cells were plated in 96-well plates at a 5×10^3^ cells/well density. AZOX was diluted with a cell culture medium to 5, 10, 15, 20, and 40 μg/ml for 48 h. 10% CCK8 solution was added to each well and incubated for 2 h, and then optical density (OD) values were measured by a microplate reader at 450 nm. IC50 value was calculated by probit regression using SPSS software.

#### Apoptosis analysis

The FITC Annexin V apoptosis detection kit I (BD, United States) was used for apoptosis analysis. DOK cells were treated with AZOX at 0, 5, 10, and 20 μg/ml for 48 h, then incubated with FITC Annexin V for 15 min and PI for 5 min in the dark. For flow cytometry analysis, a gate was set up to include only live cells and 10,000 cells per group were collected for analysis. Early apoptosis (Annexin V+/PI- cells) rates were analyzed using flowjo software.

#### Western blot

Total proteins were extracted from cells following standard protocol. Protein concentration was measured using the Lowry method. Protein samples were separated by SDS–polyacrylamide gel electrophoresis and transferred onto the nitrocellulose membrane. The membrane was then blocked and incubated with primary antibodies against Bcl-2 (1:2000, ab182858, Abcam), Bax (1:1000, ab32503, Abcam), PI3K (1:500, A4992, Abclonal), AKT antibody (1:2000, 10176-2-AP, ProteinTech), p-ERK (1:2000, #4370, Cell Signaling Technology), p-JNK (1:1000, #4668, Cell Signaling Technology), p-p38 (1:1000, #4631, Cell Signaling Technology) and VCL (1:1000, #13901, Cell Signaling Technology) antibody. Then the membrane was incubated with horseradish peroxidase (HRP)-conjugated secondary antibodies (1:2500, Pierce, United States) for 1 h. The signals were visualized using an enhanced chemiluminescence detection system.

#### Cellular thermal shift assay

DOK cells were treated with 10 μg/ml AZOX for 1 h at 37°C under 5% CO_2_ in a humidified atmosphere, then suspended in PBS with protease inhibitors. The cell suspensions in PBS were distributed into nine PCR tubes and heated to indicate temperatures (43, 46, 49, 52, 55, 58, 61, 64, and 67°C) for 3 min and immediately snap-frozen in liquid nitrogen. Samples were subjected to three freeze-thaw cycles and centrifuged at 12000 g/min for 20 min. Supernatants were used for Western blot analysis.

### Statistical analysis

The experiments were conducted in triplicate. Statistical analysis was performed using the SPSS software (IBM SPSS 26.0, United States). Differences with *p* values of less than 0.05, as analyzed by ANOVA approaches or Fisher’s exact test, were considered significant.

## Results

### Screening of potential targets

**FIGURE 1:**
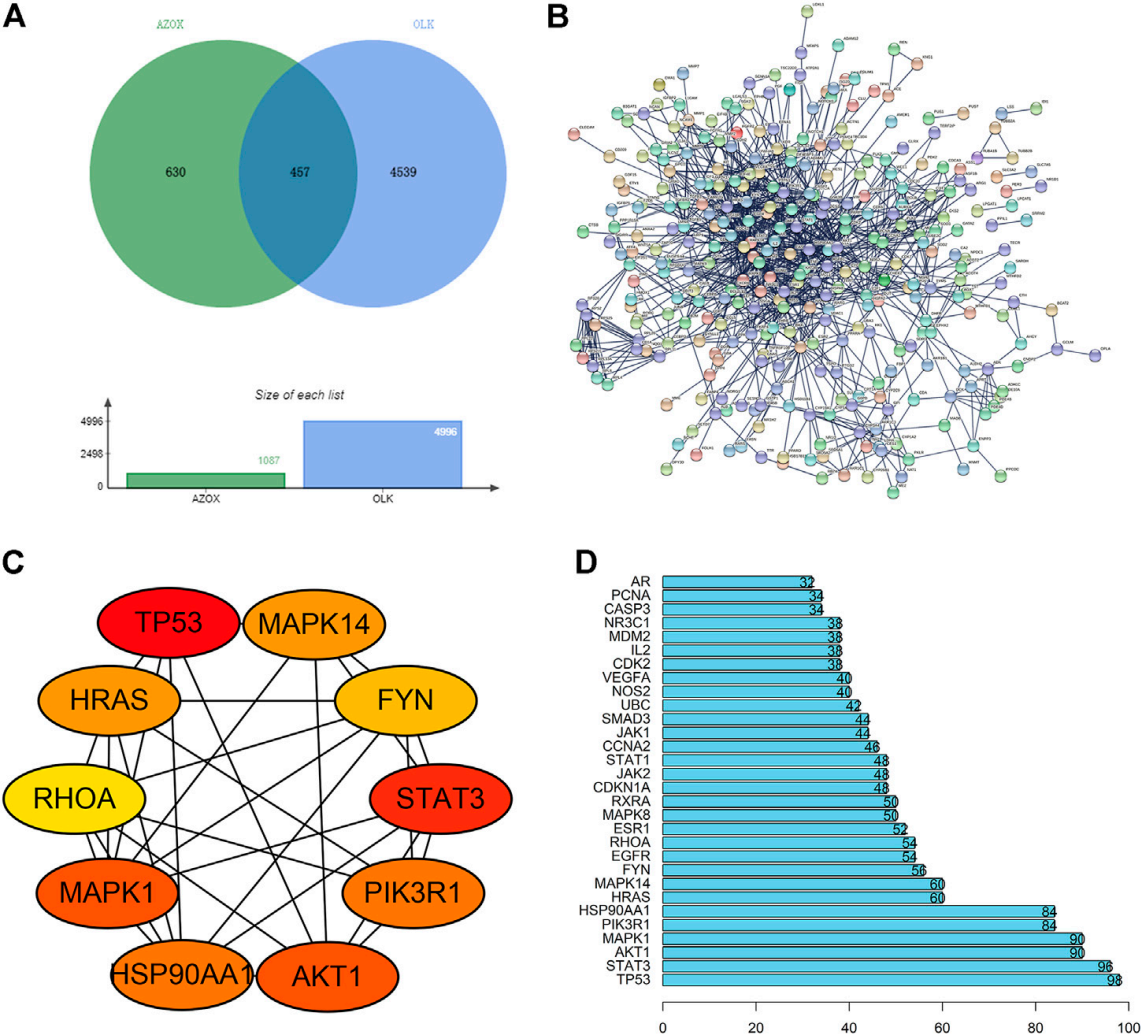
The process of screening core targets. **(A)** Venn diagram of the AZOX and OLK targets. **(B)** PPI network of overlapping genes (the potential targets) related to both OLK and AZOX. **(C)** The interaction network of top ten hub genes. **(D)** The top 30 genes selected from the PPI network of intersection genes according to the node degree.

We screened OMIM, GeneCard, and CTD datasets to identify 4539 OLK-associated targets. And 630 potential targets of AZOX were collected from the Pharmmapper online database, Swiss target prediction database, and CTD. Finally, we obtained 457 potential overlapping genes (the potential targets) related to both OLK and AZOX from the Venn diagram (Figure 1A).

### PPI and hub genes of AZOX against OLK

According to the prediction results of the STRING, the interaction between proteins was visualized, including 321 nodes and 2240 edges (Figure 1B). The top 10 hub genes were screened out according to the degree of nodes, including TP53, STAT3, AKT1, MAPK1, PIK3R1, HSP90AA, MAPK14, HRAS, FYN, and RHOA by CytoHubba. The PPI between these proteins is shown in Figure 1C. The node degrees of TP53, STAT3, AKT1, MAPK, and PIK3R1 ranked top 5 among these genes, which are 98, 96, 90, 90, and 84, respectively (Figure 1D). It is suggested that TP53, STAT3, AKT1, MAPK1, and PIK3R1 may be critical targets for AZOX against OLK.

### Biological function analysis of the potential targets

**FIGURE 2:**
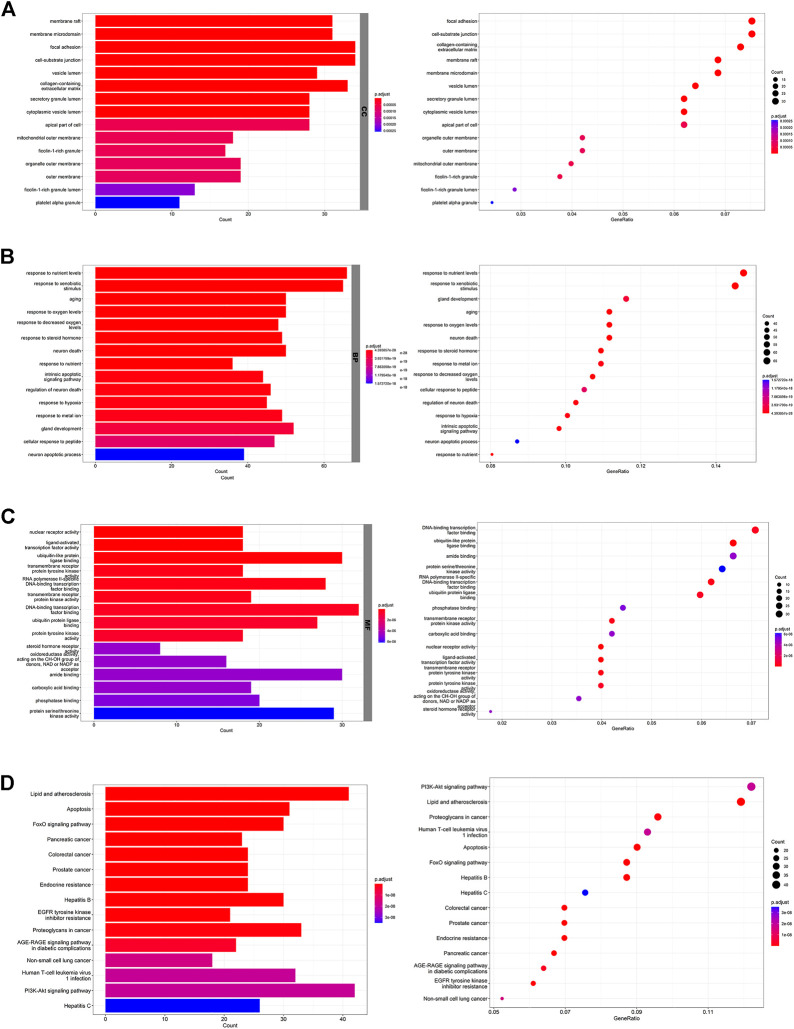
Biological function analysis of the potential targets. **(A)** The top 15 GO terms in the CC category. **(B)** The top 15 GO terms in the BP category. **(C)** The top 15 GO terms in the MF category. **(D)** The top 15 KEGG pathways.

The top 15 GO enrichment terms, including three categories: cellular component (CC), biological process (BP), and molecular function (MF), were listed in Figures 2A–C. The enriched GO terms for target genes included the membrane raft, membrane microdomain, focal adhesion, cell-substrate junction, and vesicle lumen in the CC category. Response to nutrient levels, response to xenobiotic stimulus, aging, response to oxygen levels, and response to decreased oxygen levels were in the BP category. In addition, MF involved DNA-binding transcription factor binding, ubiquitin-like protein ligase binding, amide binding, protein serine/threonine kinase activity, RNA polymerase specific DNA-binding transcription, ubiquitin-protein ligase binding, and phosphatase binding. KEGG signaling pathway enrichment results are shown in Figure 2D. The top enriched signaling pathways were mainly related to the “PI3K/AKT signaling pathway”, “MAPK signaling pathway”, and “Apoptosis”.

### AZOX inhibits the malignant transformation of tongue OLK in mice

**FIGURE 3:**
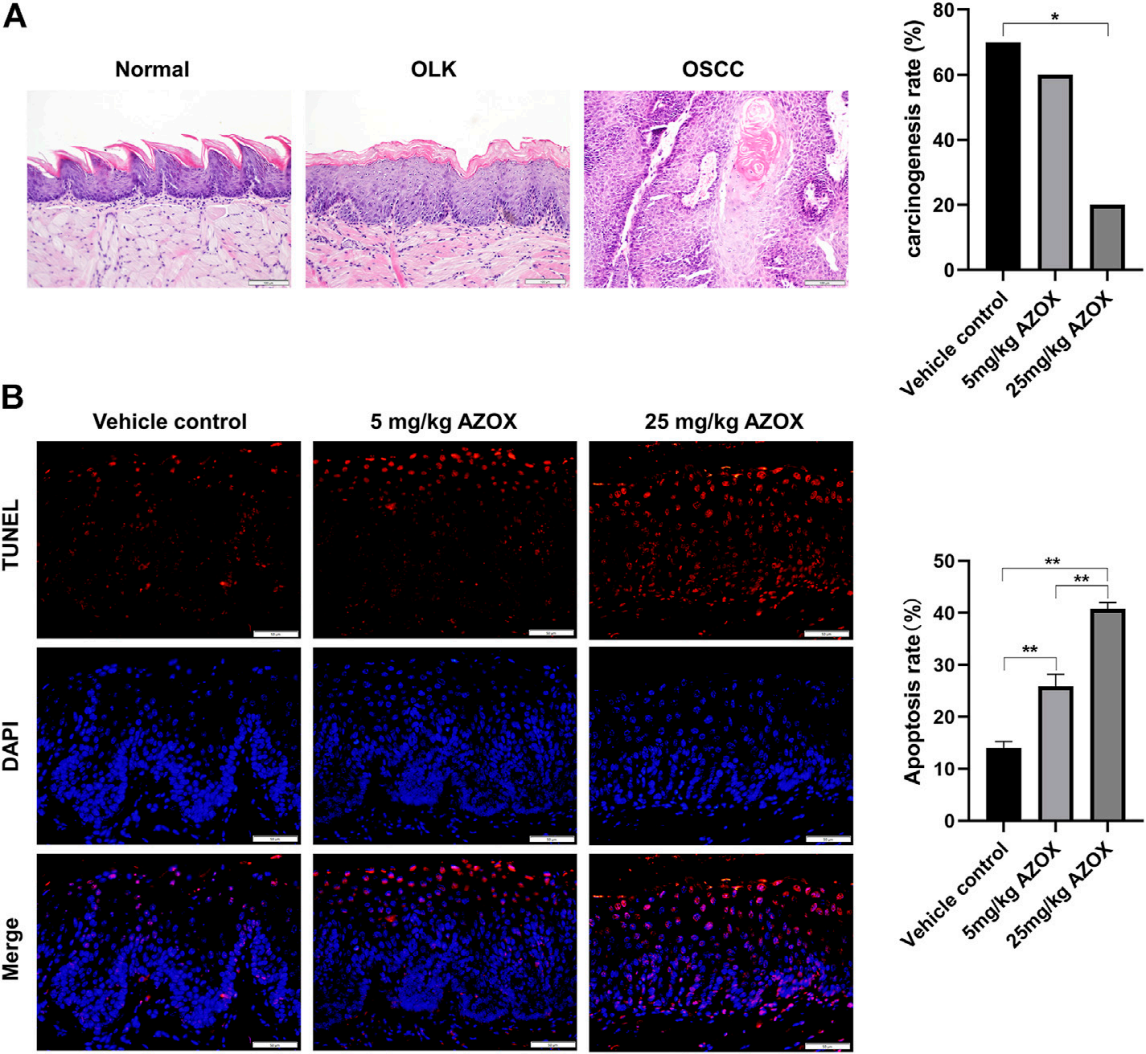
AZOX inhibits the malignant transformation and induces apoptosis in mouse OLK tissues. **(A)** Histopathology of the tongue tissues in mice (magnification 200 × ), and the carcinogenesis rate in the vehicle control group, low- and high-dose AZOX group. **(B)** Representative images of TUNEL staining results in the vehicle control group, the low- and high-dose AZOX group (magnification 400 × ), and the relative apoptosis rate was calculated using ImageJ software and shown as a bar graph. *p < 0.05, **p < 0.01.

The 4NQO-induced tongue carcinogenesis model was established in this study. The pathological diagnosis of the mouse tongue tissue in each group was shown in Table 1. The malignant transformation rate in the high-dose group (20%) was significantly lower compared with the vehicle control group (70%) (Figure 3A), which suggests that AZOX inhibits 4NQO-induced mouse tongue carcinogenesis.

**TABLE 1:** Pathological diagnosis of mouse tongue tissue in each group.

	Blank control (*n* = 4)	Vehicle control (*n* = 10)	Low-dose AZOX (*n* = 10)	High-dose AZOX (*n* = 10)
Normal	4	0	0	0
OLK with mild or moderate dysplasia	0	1	2	7
OLK with severe dysplasia	0	2	2	1
OSCC	0	7	6	2

### AZOX induces apoptosis *via* PI3K/AKT and MAPK pathways in mouse OLK tissues

**FIGURE 4:**
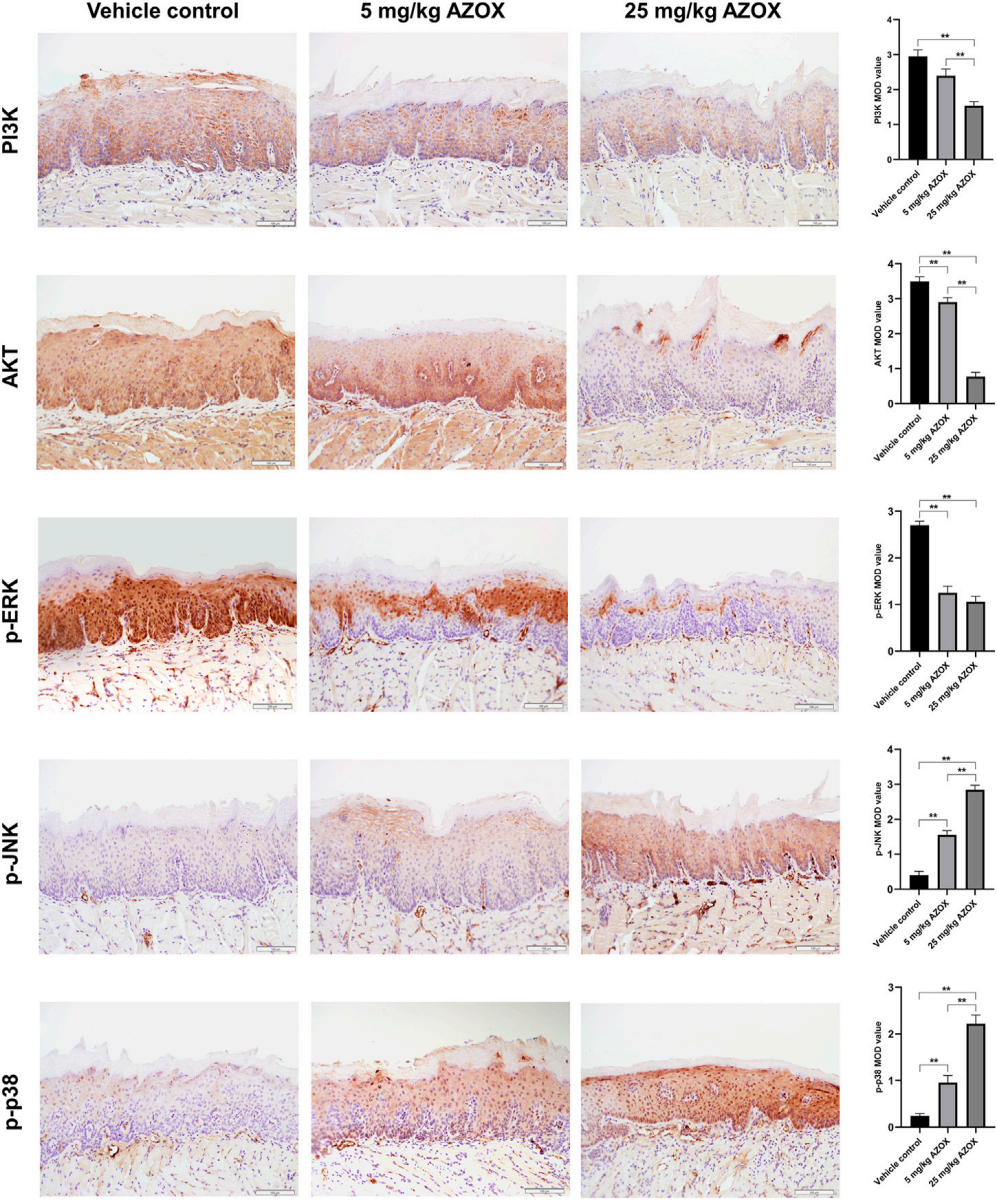
AZOX induces apoptosis *via* PI3K/AKT and MAPK pathways in mouse OLK tissues. The expression of PI3K, AKT, and MAPK pathway relative markers p-ERK, p-JNK, p-p38 was measured in mouse OLK tissues of the vehicle control group, the low and high-dose AZOX group by IHC (magnification 200 × ). The IHC images were calculated using Image-Pro Plus software 6.0 and the results were shown as bar graphs. **p < 0.01.

The apoptosis rate was significantly higher in the mouse OLK tissues in low- and high-dose AZOX groups than those in the vehicle control group by TUNEL assay (Figure 3B). IHC staining showed that the expression of PI3K, AKT, p-ERK in the OLK tissues was significantly lower in the low- or high-dose AZOX groups than those in the vehicle control group, and the expression of p-p38 and p-JNK was significantly higher in the low- and high-dose AZOX groups than those in the vehicle control group (*p* < 0.01) (Figure 4). It suggests that AZOX may induce apoptosis *via* PI3K/AKT and MAPK pathways in OLK.

### AZOX inhibits the cell viability of DOK cells

**FIGURE 5:**
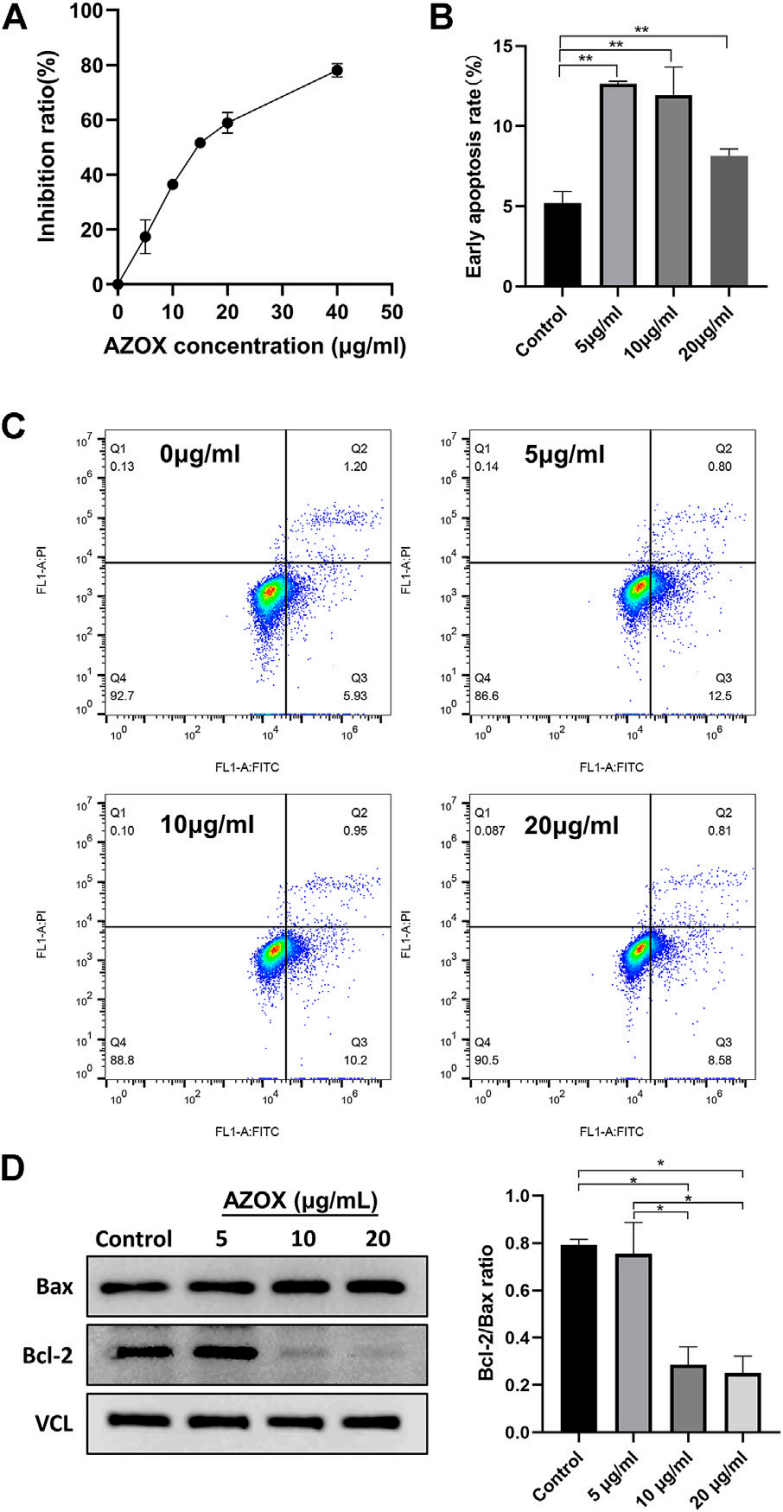
AZOX inhibits proliferation and induces apoptosis in DOK cells. **(A)** CCK8 assay was used to investigate the effect of AZOX on the activity of DOK cells. **(B)** Bar graph showing flow cytometry analysis of early apoptosis rate of DOK cells. **(C)** Early apoptotic DOK cells were examined through flow cytometry. **(D)** Western blot for the expression of Bax and Bcl-2 in DOK cells after being treated with AZOX at 0, 5, 10, 20 μg/ml for 48 h, and quantitative analysis for Bcl-2/Bax ratio. *p < 0.05, **p < 0.01.

CCK8 assay was used to investigate the effect of AZOX on the activity of DOK cells. DOK cells were treated with a series of concentrations (5, 10, 15, 20, and 40 μg/ml) of AZOX for 48 h, As shown in Figure 5A, AZOX inhibited the cell viability of DOK cells in a concentration-dependent manner, and the IC50 of AZOX was 37.3 μM.

### AZOX induces apoptosis *via* MAPK and PI3K/AKT pathways *in vitro*

**FIGURE 6:**
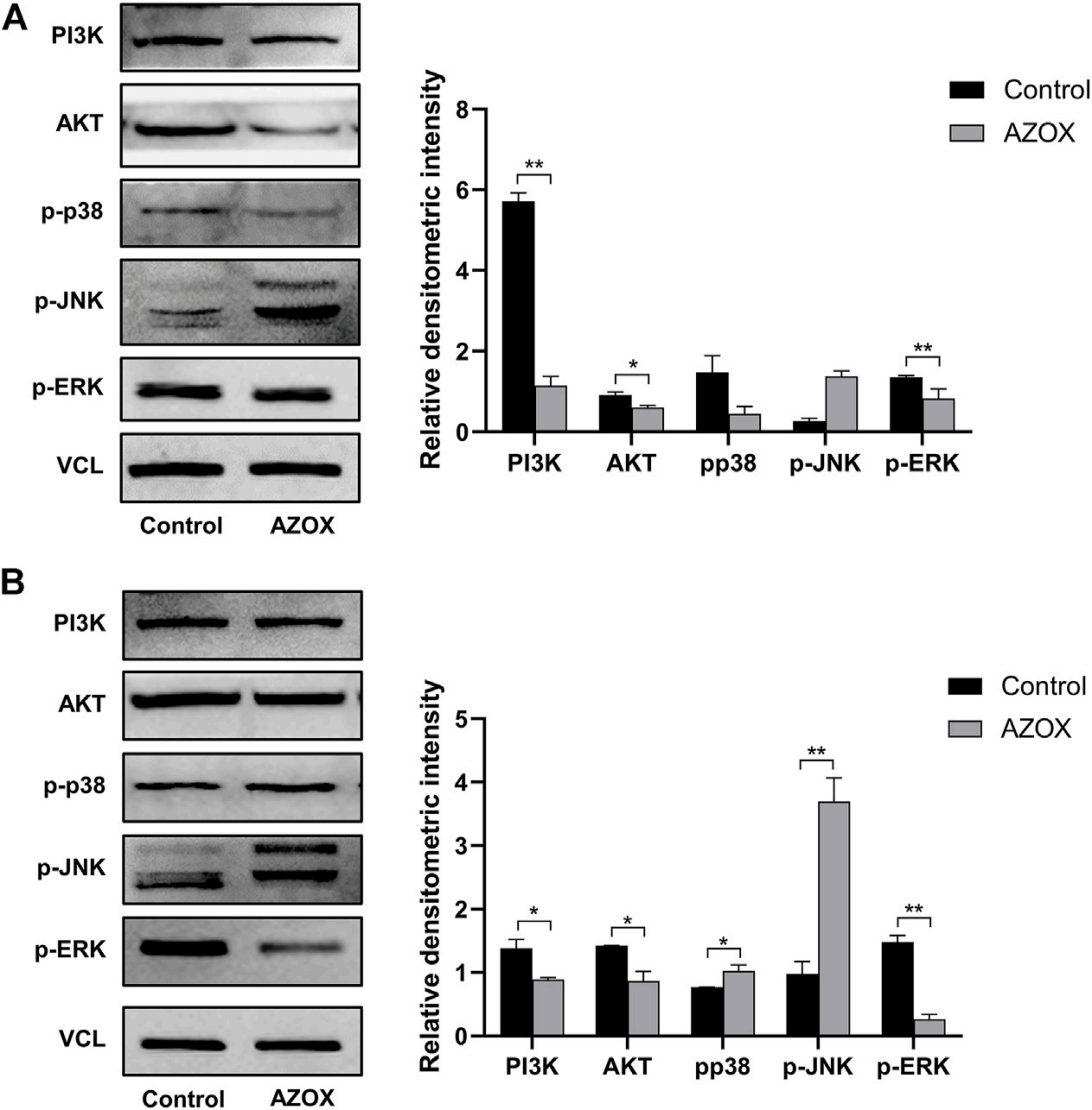
AZOX induces apoptosis *via* MAPK and PI3K/AKT pathways in DOK cells. **(A)** The expression of PI3K, AKT, and MAPK pathway relative markers p-ERK, p-JNK, p-p38 in DOK cells after being treated by 10 μg/ml AZOX for 24 h **(B)** The expression of PI3K, AKT, and MAPK pathway relative markers p-ERK, p-JNK, p-p38 in DOK cells after being treated by 10 μg/ml AZOX for 48 h. *p < 0.05, **p < 0.01.

After being treated with 0, 5, 10, and 20 μg/ml AZOX for 48 h, the early apoptosis rate of DOK cells treated with AZOX increased significantly by flow cytometry (*p* < 0.01) (Figures 5B,C). And 10 ug/mL and 20 ug/mL had a more significant effect on the expression level of apoptosis-related molecules (Bcl2/Bax ratio) in DOK cells after being treated with 0, 5, 10, and 20 μg/ml AZOX for 48 h (Figure 5D). We thus chose 10 ug/ml of AZOX to explore the mechanism underlying AZOX-induced DOK cell apoptosis. As the results displayed in Figure 6A, after being treated with 10 μg/ml AZOX for 24 h, a significantly decreased expression of PI3K, AKT, p-ERK in DOK cells was observed compared with the control group (*p* < 0.05 or *p* < 0.01). Compared to the control group, the expression of PI3K, AKT, p-ERK was significantly decreased in DOK cells treated with 10 μg/ml AZOX for 48 h (*p* < 0.05 or *p* < 0.01), and the expression of p-p38 and p-JNK was significantly increased in DOK cells treated with 10 μg/ml AZOX for 48 h (*p* < 0.05 or *p* < 0.01) (Figure 6B).

### Comparison of hub targets by molecular docking and verification

**FIGURE 7:**
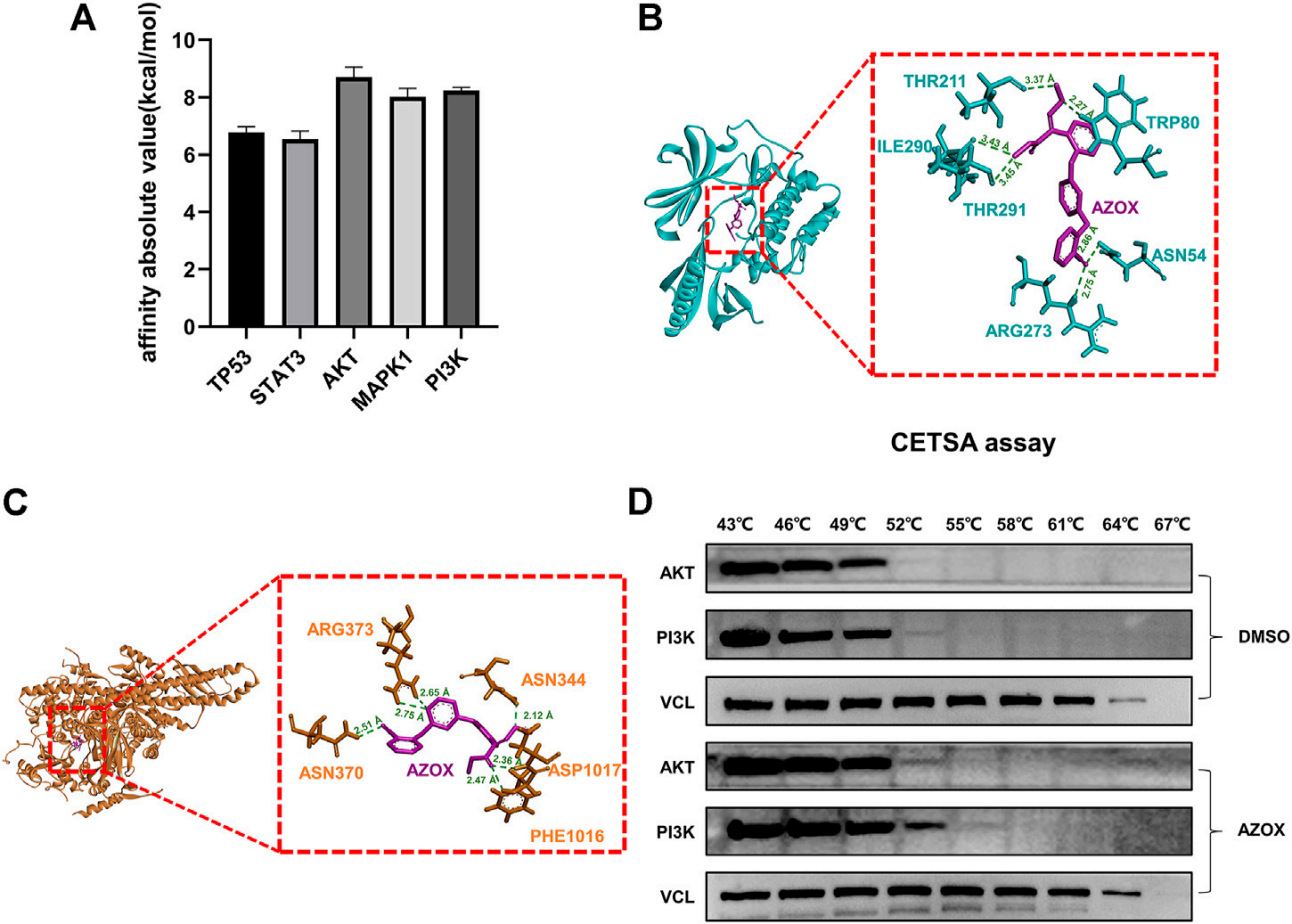
Comparison of hub targets by molecular docking and verification. **(A)** Bar graph of hub protein mean score values of the top ten docking scores. **(B)** 3D docking conformation of AZOX with AKT. Green dashed lines represent H-bonds and the numbers denote the distance of the H-bonds. **(C)** 3D docking conformation of AZOX with PI3K. Green dashed lines represent H-bonds and the numbers denote the distance of the H-bonds. **(D)** CETSA and Western blot analysis showed that AZOX may directly bind to AKT and PI3K.

AZOX and the top five hub target proteins were prepared by AutoDockTools 1.5.6 software. Molecular docking was performed using AutoDock Vina. The top ten docking scores of AZOX with target proteins were selected. The obtained results were ranked according to their mean score values of the top ten docking scores and presented in kcal/mol (Figure 7A). After calculations, we found that AKT, PI3K were ranked in the top two, indicating that AKT and PI3K might have more related to AZOX.

To further investigate the docking residue sites of AZOX and the target proteins, PyMOL software was used to visualize the corresponding protein residues in a line shape. As shown in Figure 7B, the hydrogen bonds were formed between AZOX and AKT residue THR211 (distance of 3.37 Å), ILE290 (distance of 3.43 Å), TRP80 (distance of 2.27 Å), THR291 (distance of 3.45 Å), ASN54 (distance of 2.86 Å), and ARG273 (distance of 2.75 Å). Moreover, AZOX interacted with TRP-80 by a *pi*-*pi* interaction. The hydrogen bonds were formed between AZOX and PI3K residue PHE-1016 (distance of 2.47 Å), ASP1017 (distance of 2.36 Å), ASN-344 (distance of 2.12 Å), ASN370 (distance of 2.51 Å), and ARG373 (distance of 2.75 Å and 2.65 Å) (Figure 7C).

In addition, we also performed cellular thermal shift assay (CETSA) further to verify the interaction between AZOX with AKT and PI3K. The results showed that as the temperature rose, PI3K and AKT became more stable in DOK cells treated with AZOX compared with the control group, which indicated that AZOX might directly bind to AKT and PI3K in OLK (Figure 7D).

## Discussion

OSCC is the sixth most common tumor, with a higher mortality rate and its 5-years survival rate is 45-60% (Vaidya et al., 2022). Of note, early detection and effective treatment have priority to reduce lethality. OLK is one of the most common OPMDs worldwide, and the carcinogenesis rate of OLK varies from 0.13 to 34% (Jayasooriya et al., 2020). Although there are diverse intervention methods for OLK, the effects are not satisfactory because of its irreversible progression (Hogewind et al., 1989).

Natural products play a particularly significant role in the cancer prevention, such as lycopene, curcumin, aloe vera, green tea, and so on (Salehi et al., 2019; Ahmad et al., 2021). AZOX, as a natural product, its studies have primarily focused on the role of crop and aquaculture. A recent toxicological study of AZOX in mammals confirmed that AZOX has minimal toxic effects in mammals, including rats and humans (Imura et al., 2019). AZOX inhibits the fungus mitochondrial respiration and induces oxidative stress *via* a block of the electron transfer between cytochrome b and cytochrome c1 (Bartlett et al., 2002; Han et al., 2016). AZOX affects cell proliferation and growth in fish (Olsvik et al., 2010). In the aquatic macrophyte Myriophyllum quitense, 50 μg/L AZOX can significantly inhibit the antioxidant enzyme systems, and 50 and 100 μg/L AZOX can result in lipid and DNA damage (Garanzini and Menone, 2015). However, the therapeutic potential of AZOX in mammals has been poorly investigated. In our previous studies, anti-tumor active components were screened out of 70 natural plant products, and we found that AZOX induces apoptosis, inhibits cell proliferation and the activity of mitochondrial complex in OSCC cell line SCC15 and CAL27 cells, and decreases the incidence of 4NQO-induced tongue OSCC in mice (Chen et al., 2020). This study found that AZOX inhibited cell viability in an obvious concentration-dependent manner. And AZOX inhibited the malignant transformation of tongue OLK in mice. For the future potential use of AZOX in clinical setting for treatment of OLK, further investigation of the effects of AZOX on different clinical and histological severity of OLK lesions should be performed in a follow-up study.

Network pharmacology exerts a valuable tool for rapidly screening drugs and targets and action mechanisms of drugs, significantly reducing drug development time and cost. Besides, it can systematically analyze the interaction between drugs and diseases. By establishing a “compound-protein/gene-disease” network, it reveals the regulatory principles of small molecules in a high-throughput manner (Zhang et al., 2019). In this study, network pharmacology was used to predict the potential targets of AZOX in the treatment of OLK. We harvested 457 potential targets related to AZOX and OLK from public databases. The interaction among the targets was obtained by constructing the PPI network, including 321 target genes and 2240 interactions. Here five hub genes were selected for further analysis using the plugin cytoHubba in Cytoscape, including TP53, STAT3, AKT1, MAPK1, and PIK3R1. TP53 is a tumor suppressor gene that could induce apoptosis, cell cycle arrest, and senescence. It has been shown that AZOX induces apoptosis and cell cycle arrest in p53-negative human myelogenous leukemia cell line HL-60RG and the p53 positive human T-cell leukemia cell line MOLT-4F (Takahashi et al., 2022). Over-expression of p53 occurs in early oral carcinogenesis resulting in defective apoptosis and subsequent tumor progression (Ravi et al., 1996). STAT3, identified as an oncogene, has proved to regulate apoptosis, cell proliferation, angiogenesis, and the immune response. STAT3 inhibits apoptosis by regulating apoptosis-related gene expression during the development of tumors. STAT3 activation is an early event in the progression of low-grade dysplasia to OSCC (Crawford et al., 2021). MAPK1, also known as ERK1 or ERK2, is a prominent MAPK member, the crucial protein to transmit the signals from surface receptors to the nucleus. The phosphorylation activated ERK1/2 is involved in apoptosis, cell proliferation and differentiation, and malignant transformation of cells. The expression of p-ERK was significantly higher in high-grade epithelial dysplasia than in OLK, suggesting that ERK might play an essential role in oral carcinogenesis (Tashiro et al., 2020). PI3KR1 codes for PI3K regulatory subunit alpha; while AKT is the primary downstream pathway gene of PI3K, they are anti-apoptosis factors. Studies confirmed that activating the PI3K-AKT signal pathway is involved in oral carcinogenesis (Watanabe et al., 2009; Tashiro et al., 2020). It suggests that the five hub genes are related to apoptosis in OLK, and AZOX can induce apoptosis in OLK. This study confirmed that the early apoptosis rate increased significantly in DOK cells treated with AZOX by flow cytometry. And the apoptosis rate was significantly higher in the mouse OLK tissues in low- and high-dose AZOX groups than those in the vehicle control group by TUNEL assay. Research has shown that AZOX may target the mitochondria, induce cytochrome c expression, and activate caspase-3 by the Bax channel, leading to the mitochondrial pathway of apoptosis in KYSE-150 cells (Shi et al., 2017). These data suggest that AZOX inhibits the malignant transformation by inducing apoptosis in OLK.

In the present study, the biological functions of these genes were further investigated. The enriched GO functions involved DNA-binding transcription factor binding, ubiquitin-like protein ligase binding, amide binding, protein serine/threonine kinase activity, RNA polymerase specific DNA-binding transcription, ubiquitin-protein ligase binding, and phosphatase binding. In KEGG analyses, a variety of signaling pathways were enriched, PI3K-AKT signaling pathway, MAPK signaling pathway, and apoptosis were key signaling pathways among them. Studies have revealed that PI3K/AKT and MAPK pathways play important roles in cell apoptosis. However, the specific roles of AZOX on PI3K/AKT and MAPK signal pathways in OLK need further confirmation. To verify the predicted results of network pharmacology, we performed corresponding *in vivo* and *in vitro* experiments. By establishing the 4NQO-induced tongue carcinogenesis model, we found that 25 mg/ml AZOX significantly decreased the incidence of tongue cancer in mice. AZOX induced apoptosis in mouse OLK tissues. Compared with the vehicle control group, the expression of PI3K, AKT, p-ERK was significantly lower in the low- and high-dose AZOX groups. The expression of p-p38 and p-JNK was significantly higher in the low- and high-dose AZOX groups. Additionally, AZOX inhibited proliferation and induced apoptosis in DOK cells. After treated with AZOX for 24 and 48 h, a significantly decreased expression of PI3K, AKT, p-ERK was observed in DOK cells. And a significantly increased expression of p-p38 and p-JNK was observed after treated with AZOX for 48 h in DOK cells. Our previous studies also showed that nicotine, mainly a risk factor for OLK pathogenesis, suppresses apoptosis by regulating the α7nAChR/Prx1 axis and suppressing MAPK activation in oral precancerous lesions. Collectively, these data suggest that AZOX may induce apoptosis via PI3K/AKT and MAPK signal pathways in OLK.

To further investigate the mechanisms of AZOX in OLK, we compared the hub targets by molecular docking and found that the binding force of AZOX and AKT ranked was the strongest, followed by PI3K. PyMOL software was used to visualize the corresponding protein residues in a line shape. The hydrogen bonds were formed between AZOX and AKT residue THR211, ILE290, TRP80, THR291, ASN54, and ARG273. Besides, AZOX could interact with TRP-80 by a *pi*-*pi* interaction. AZOX hydrogen-bonded with PHE-1016, ASP1017, ASN-344, ASN370, and ARG373 on PI3K. CETSA is a biophysical technology that can directly determine the combination of drugs and target proteins in cells and tissues by thermal stability changes under heating conditions (Martinez Molina et al., 2013). As the protein bind to the drug, the composite protein will be more stable at the same temperature. Thus, in the current study, we performed CETSA to further test and verify the interaction between AZOX and AKT or PI3K. The results showed that with the rise of the temperature, AKT and PI3K were proved to be more stable in DOK cells treated with AZOX. Our data indicate AZOX directly binds to AKT and PI3K to active PI3K/AKT and MAPK signal pathways, then induces apoptosis in OLK.

## Conclusion

This study, for the first time, applies network pharmacology and molecular docking technology to investigate the roles and mechanisms of AZOX in OLK. It suggests that AZOX may induce apoptosis *via* PI3K/AKT and MAPK pathways in OLK. AZOX has the potential to develop into a new drug for the treatment of OLK. However, further studies are needed to be devoted to the binding sites of AZOX with key targets in OLK.

## Data Availability

The original contributions presented in the study are included in the article/Supplementary material, further inquiries can be directed to the corresponding author.
